# Anatomy of Antisemitism in Health Professions Education and Practice: A Narrative Review

**DOI:** 10.5041/RMMJ.10581

**Published:** 2026-07-31

**Authors:** Hedy S. Wald, Steven Roth

**Affiliations:** 1 Department of Family Medicine, Warren Alpert Medical School of Brown University, Providence, Rhode Island, USA; 2 Department of Anesthesiology, College of Medicine, University of Illinois, Chicago, Illinois, USA

**Keywords:** Antisemitism in medicine, antisemitism in healthcare professions, medicine during the Holocaust, professional identity formation

## Abstract

There has been an international resurgence in antisemitism in healthcare education and practice since October 7, 2023, prominently affecting the United States (US), United Kingdom (UK), Canada, and Australia. In the US, federal investigations by the US Department of Justice and the Congressional Subcommittee on Education and the Workforce were initiated into antisemitic harassment and hostile learning environments in medical schools. Healthcare organizations in the UK also came under investigation, and the US Congress opened an investigation into alleged antisemitism within a national psychological association. Quantitative and descriptive studies of the prevalence and characteristics of this phenomenon within medical, psychology, and social work education and practice are scant. We conducted a narrative review given the under-recognized nature of antisemitism in this context and the limited literature on this international, inter-professional issue. We searched PubMed using a search string to capture peer-reviewed descriptive and quantitative studies of antisemitism in medicine, psychology, social work, nursing, pharmacy, physician assistant, dentistry, physical, and occupational therapy education and practice. We searched 17 ProQuest databases for media and other online or written materials and conducted a hand search for additional relevant publications. We identified studies showing a high prevalence of antisemitism particularly in medical education, psychology, and social work, where >50% of subjects experienced antisemitism since October 7, 2023. Fewer than 2% of institutions had incorporated education about antisemitism into anti-discrimination training. Our review revealed that antisemitism as a form of bigotry, hatred, and discrimination has been inadequately addressed, now taking on urgency given its reach including an anti-Israel bias “mutation” and the need to assure unbiased healthcare. Actionable recommendations to counter antisemitism in healthcare derived from the publications to date are presented.

“If we want to fight antisemitism, let us walk tall and proud as Jews, and let us work with all humanity to banish hatred forever.”

*Rabbi Lord Jonathan Sacks* 1

## INTRODUCTION

Graduating physicians take oaths to respect sanctity of life, bring humility, and enter a covenant of service. All health profession trainees are trained to adhere to professional ethics charters. These oaths and charters are challenged by contradictions posed by antisemitism in healthcare, particularly its educational component, which surged after the October 7, 2023 Hamas terrorist attack on Israel.^2^ The moral imperative to counter antisemitism in medicine is inclusive of medical education and professional conduct. Advocacy against hate is a core component of medical professionalism.^2^ The health professions have challenged past and ongoing injustices. Antisemitism is no exception and deserves attention—there is work to be done in countering all forms of discrimination. This includes antisemitism in the health professions, albeit challenging as an under-recognized prejudice and because antisemitism has adapted over the millennia, leading to its label as a “mutating virus”^3^ that constantly changes its structure. The history of this virus through the millennia, adapting to the zeitgeist, includes hate on religious grounds, then shifting to racial hate (leading to the horror of the Holocaust), and then to anti-Israel hate/demonization or an anti-Zionist form.^3^ Elie Wiesel taught about harm of bystander silence, of the inherent complicity.^4^ Antisemitism is often described as a “canary in the coal mine,” a predictor of wider societal decay and bigotry.^5^ Attitudes and behavior not tolerated for other groups, therefore, cannot be tolerated for Jews.

We identified the need for a narrative review given the above and this international and inter-professional issue not being extensively described. Federal investigations into antisemitic harassment constituting hostile learning environments in medical schools^6^,^7^ have recently been initiated in the United States (US) as well as investigations of rampant antisemitism within a national psychological association.^8^ Federal Bureau of Investigation (FBI) statistics showed a 5.8% increase in hate crimes in 2024 versus 2023 against Jews, the highest number or percent yearly increase recorded.^9^ Religion-related hate crimes in the US disproportionately impact Jews (2% of the population), representing 16% of reported hate crimes and 70% of religion-based 2024 hate crimes.^9^ While Jews make up approximately 10% of the population of New York City, >50% of hate crimes were committed against Jews in the first quarter of 2026.^10^ In a US Jewish community 2025 survey, 55% had experienced at least one form of antisemitism in the last 12 months, antisemitism had become a “normal Jewish experience” for 57%, while 79% expressed concerns about antisemitism, and 48% reported having taken actions to increase personal security and safety.^11^ In Europe and Australia, since October 7, 2023, there has been a 400% rise in hate incidents and violence targeting Jews.^12^ The United Kingdom (UK) Community Security Trust reported a 324% increase in antisemitic incidents between October 7 and 10, 2023 versus the same period the year before and a 413% increase in incidents in universities in the year following the Hamas October 7, 2023 atrocities.^13^

Antisemitism in health professions education (HPE) is not new. Jewish quotas for admission to US and Canadian medical, dental, and psychology schools, and medical residency training, were widespread during the first half of the 20th century, in response to Jewish immigration from the 1880s to 1920.^14^,^15^ Today we witness the resurgence of global antisemitism which has bled into the healthcare professions, both in HPE and practice.

## METHODS

### Review Design

To enhance the rigor and transparency of this narrative review, relevant elements of the 2020 Preferred Reporting Items for Systematic Reviews and Meta-Analyses (PRISMA) guidelines were used to inform the reporting of the literature search and source-selection process.^16^

Structured searches were performed in PubMed and ProQuest, and then supplemented by hand-searching relevant sources. The search for literature and other relevant materials was limited to English-language sources published between October 7, 2023 and March 6, 2026.

PubMed^17^ and ProQuest^18^ were searched to identify relevant peer-reviewed publications and media reports, and other online and printed materials, respectively. Additional records were identified through hand search of citations from identified articles and reviews. Although social media were not searched directly, eligible publications that analyzed social-media data were included. The International Holocaust Remembrance Alliance (IHRA) definition of antisemitism was used as an objective reference standard, as described elsewhere.^19^,^20^ By adopting this definition, the authors were able to apply explicit criteria when assessing and categorizing the manifestations of antisemitism described in the included sources.^20^

### Search Strategy: PubMed

#### PubMed search filters

PubMed was searched for peer-reviewed scholarly literature using a single search string (Table 1) to capture the major healthcare disciplines. The truncator (*) captured variants of antisemitism, antisemitic, antisemite. Field-specific tags ([All Fields]) ensured comprehensive retrieval across titles, abstracts, and indexed terms. This search returned 61 records, which after applying inclusion/exclusion criteria yielded the final set of 23 results.

**Table 1:** Search Strings Used.

Database	Search Strings Used
**PubMed** for peer-reviewed scholarly literature	(antisemit*[All Fields]) AND (“medicine”[All Fields] OR “medical student”[All Fields] OR “physician”[All Fields] OR “medical education”[All Fields] OR “psychology”[All Fields] OR “psychology student”[All Fields] OR “psychologist”[All Fields] OR “psychology education”[All Fields] OR “social work”[All Fields] OR “social work student”[All Fields] OR “social worker”[All Fields] OR “social work education”[All Fields] OR “occupational therapy”[All Fields] OR “occupational therapy student”[All Fields] OR “occupational therapist”[All Fields] OR “occupational therapy education”[All Fields] OR “physical therapy”[All Fields] OR “physical therapy student”[All Fields] OR “physical therapist”[All Fields] OR “physical therapy education”[All Fields] OR “physiotherapy” [All Fields] OR “physiotherapy education”[All Fields] OR “dentistry”[All Fields] OR “dental student”[All Fields] OR “dentist”[All Fields] OR “dental education”[All Fields] OR “dentistry education”[All Fields] OR “pharmacy”[All Fields] OR “pharmacy student”[All Fields] OR “pharmacist”[All Fields] OR “pharmacy education”[All Fields] OR “nursing”[All Fields] OR “nursing student”[All Fields] OR “nurse”[All Fields] OR “nursing education”[All Fields] OR “speech-language pathology”[All Fields] OR “speech-language pathology student”[All Fields] OR “speech pathology”[All Fields] OR “speech pathology education”[All Fields] OR “communication disorders”[All Fields] OR “communication disorders education”[All Fields] OR “physician assistant”[All Fields] OR “physician assistant student”[All Fields] OR “physician assistant education”[All Fields])
**ProQuest**	(antisemit*) AND (medicine OR “medical student*” OR physician* OR “medical education” OR psycholog* OR “psychology student*” OR “social work*” OR “social work student*” OR “social worker*”)

antisemit*, truncated search term for antisemite, antisemites, antisemitism; psycholog*, truncated search term for psychologist, psychology, psychological.

#### Export and deduplication

The search string process eliminated duplicates across disciplines. Performing the search with the disciplines one at a time then combining them all versus the single search string yielded the same results. Adding the medical subject headings (MeSH) term for antisemitism “prejudice” within the search yielded 20 of the final set of 23 articles. We independently screened and evaluated the records utilizing exclusion criteria, resolving any lack of agreement through discussion and reached consensus with 23 records retained (Supplementary Material). Additional records were identified through hand search citation tracking from relevant articles and reviews, yielding an additional 49 articles (Supplementary Material).

#### PubMed exclusion criteria

Exclusions were: (1) focus on historical antisemitism predating October 7, 2023; (2) non-English language; (3) not substantively relevant to healthcare or health professions education; or (4) addressed antisemitism exclusively in the context of COVID-19.

### Search Strategy: ProQuest

#### ProQuest search filters

ProQuest was searched for media reports and other online and written materials. This database also included peer-reviewed journal articles from social science databases that are not included in PubMed (Tables 2–3). The search was narrowed to the three health professions most represented in PubMed in this domain: medicine, psychology, and social work (Supplementary Material). Truncation (*) was used to detect term variants. Filters (Table 2) were applied to yield 713 records after ProQuest’s built-in deduplication.

**Table 2:** Filters used in ProQuest Database Search.

Filter	Setting
Source type	Media, government documents, newspapers, variable coverage for scholarly journals (see Table 3 for details)
Language	English only
Date range	October 7, 2023 to March 6, 2026
Full text	Full text availability required
Document types included	News; Commentary; Feature; Correspondence; Letter to Editor; Editorial; Review; General Information; Article; Front Matter
Document types excluded	Transcript; Blog; Interview; Credits/Acknowledgments

**Table 3:** ProQuest Databases Searched.

ProQuest Database	Description/Subject Coverage
ABI/INFORM Global	A ProQuest database for business, management, trade news; professional publications; SSRN working papers
American Periodicals	General interest American periodicals; broad societal and cultural coverage
Dissertations & Theses @ Big Ten Academic Alliance	Multidisciplinary dissertations and theses from Big Ten institutions
Education Research Index (ERIC; 1966–current)	Unified ERIC + Supplemental Education Index; education research literature
ERIC (1966–current)	Education and related topics; journal articles, government documents, theses, reports
Ethnic NewsWatch Collection	Ethnic and minority publications; journal articles across social sciences and humanities
History Vault	Manuscript and archival collections; digitized historical primary sources
Linguistics and Language Behavior Abstracts (1973–current)	Theoretical and applied linguistics, psycholinguistics; journal articles, dissertations
PAIS Index (1914–current)	Public affairs, social policies, international relations; gray literature and government documents
Periodicals Archive Online	Full-text archives of arts, humanities, and social sciences scholarly journals
ProQuest Dissertations & Theses Global	Multidisciplinary dissertations and theses; 6 million records from 70+ countries
ProQuest Recent Newspapers: Chicago Tribune (2008–current)	Digitized Chicago Tribune newspaper archive
PTSDpubs (1871–current)	Literature related to traumatic stress; journal articles in health and social sciences; produced by the US Department of Veterans Affairs National Center for PTSD (formerly PILOTS).
Publicly Available Content Database	Full text of publicly available content from diverse global sources
Sociological Abstracts (1952–current)	International sociology literature; includes Social Services Abstracts
US Newsstream Collection (1980–current)	US news content; top newspapers, newswires, and news sites in full text
Worldwide Political Science Abstracts (1909–current)	International political science and international relations; journal articles, dissertations

ERIC, Education Resources Information Center; PAIS, Public Affairs Information Service; SSRN, Social Science Research Network.

#### ProQuest databases

The search was executed across 17 ProQuest sub-databases in the University of Illinois at Chicago Library subscription (Table 3).

#### Export and deduplication

Due to reference-exporting limitations, ProQuest results were batch-exported, combined, and de-duplicated using article title as unique identifier. Duplicate syndicated or identical news articles were excluded during manual review.

#### Screening/inclusions/exclusions

ProQuest records were screened through manual title and brief abstract review and retained if they included the words antisemitism, Jews, or Jewish, yielding 713 articles. We then screened full text, and records were included if they met at least one of the following criteria:

1. Antisemitism + academic setting
2. Antisemitism + medicine/psychology/social work
3. Federal/congressional investigation of healthcare or medical education
4. Antisemitic incident involving a healthcare professional

Exclusions included records not meeting any of these criteria such as antisemitism in political, religious, or general contexts unconnected to healthcare or health professions education, leaving 46 final records.

## RESULTS

### Literature Search

**Figure 1:**
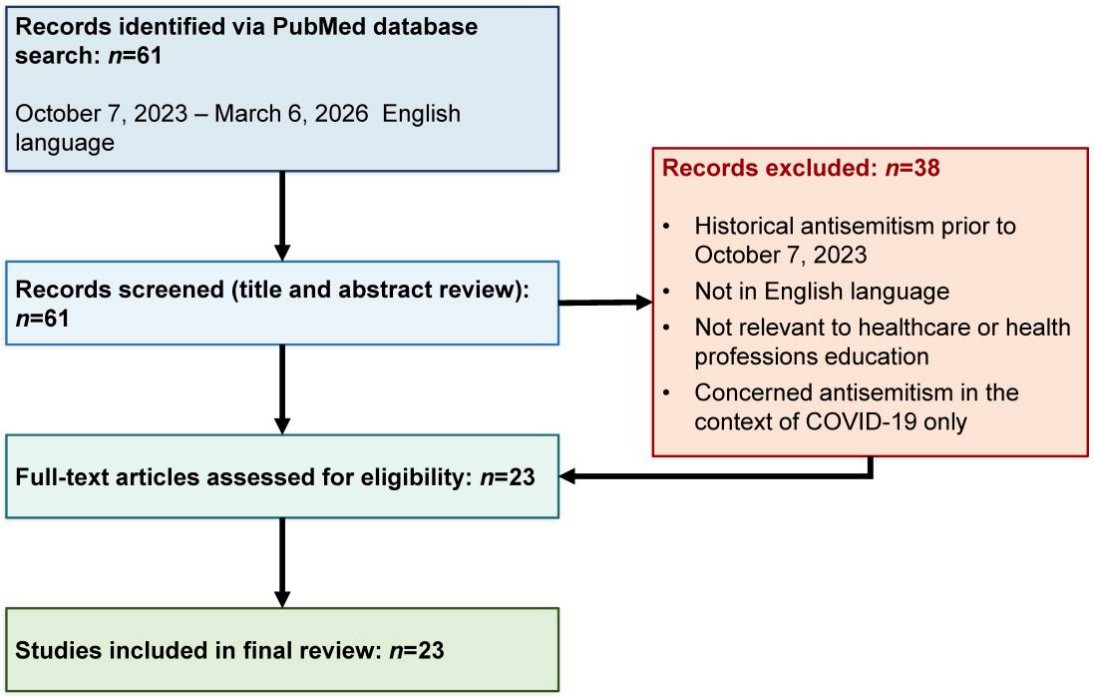
PRISMA Diagram for PubMed Search Shows the Process of Review, Inclusions, and Exclusions.

**Figure 2:**
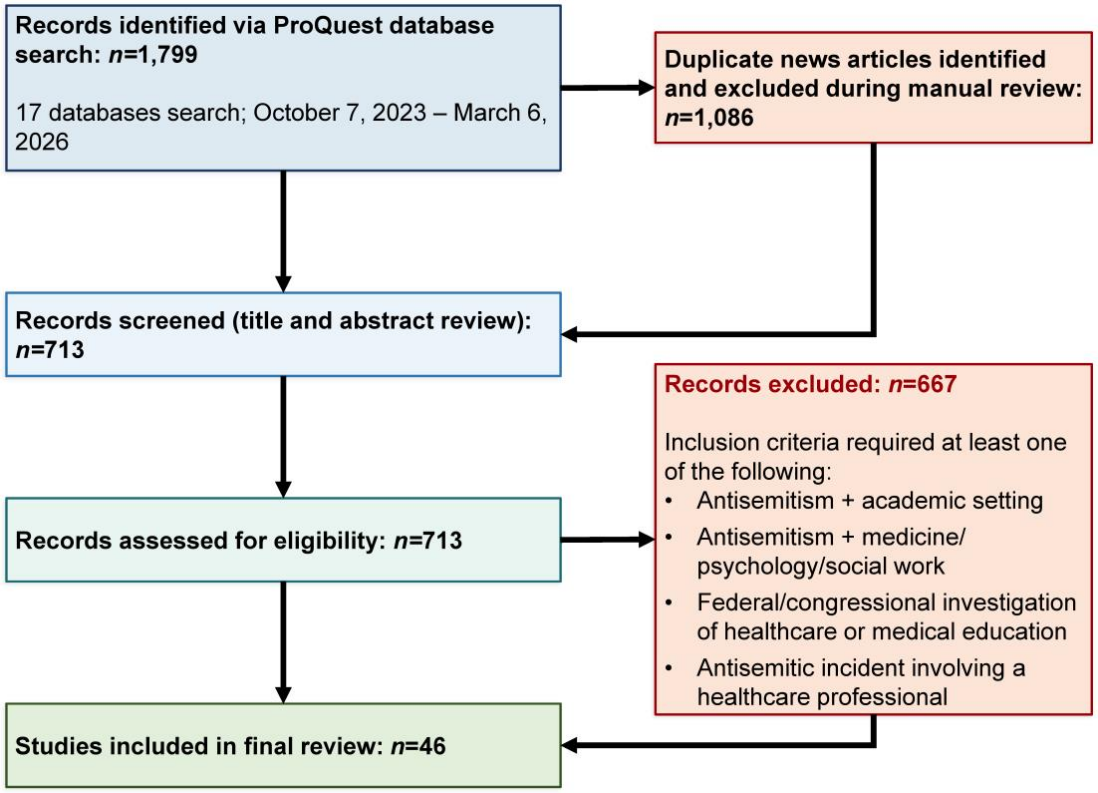
PRISMA Diagram for ProQuest Search Shows the Process of Review, Inclusions, and Exclusions.

Search for publications on antisemitism and healthcare professions in PubMed yielded 23 articles (Figure 1) and in ProQuest, 46 items (Figure 2). Additional records identified through hand search tracking yielded an additional 49 articles.

### Quantitative Evidence on Antisemitism in the Health Professions Since October 7, 2023

Quantitative evidence on antisemitism in health professions education and practice in the US, UK, Canada, and Australia is summarized in Table 4. The evidence included peer-reviewed surveys, surveys reported in media sources, an observational analysis of medical-school commencement ceremonies, and a quantitative social-media analysis.

**Table 4:** Quantitative Evidence on Antisemitism in Health Professions Education and Practice.

Field and Source(s)	Study Design, Population and Setting	Key Findings
**Medicine** Fishman and Auerbach (2026)^21^ Michelson et al. (2025)^22^ Schwartz et al. (2025)^23^ Schwartz et al. (2026)^19^	Survey of Jewish medical students and physicians in the US	Since October 7, 2023: Risk of exposure to antisemitism was four times higher in academic vs community settings^21^ <2% reported that their institutions had incorporated education about antisemitism into anti-discrimination training^22^; 88% reported experiencing antisemitism, compared with 40% before October 7^23^ >35% reported personally directed antisemitic incidents^23^ >30% reported face-to-face antisemitic encounters^23^ Nearly 50% reported exposure to antisemitism on social media; 21% reported exposure in classes or seminars^23^

**Medicine** Roth and Wald (2025)^24^	Observational review of commencement ceremonies for the year 2024 amongst the 25 highest-ranked US medical schools	The proportion of graduating students who wore specified regalia or buttons, carried signs or banners, interrupted ceremonies verbally, or deviated from approved scripts ranged from 0% to 22.5% across the medical schools studied Examples included stoles combining a Palestinian flag, a keffiyeh, and an empty map of Israel, some with the word “Palestine” next to it in Arabic, accompanied by Arabic-language slogans (“Jerusalem is Ours”), and the display of a sign reading “End Zionism, end apartheid” The authors interpreted these displays as denying the legitimacy of the State of Israel and the Jewish people’s right to self-determination

**Medicine** Gordon et al. (2025)^25^	Cross-sectional electronic survey of 265 Jewish healthcare students and professionals in Victoria, Australia, conducted from 12/2023 to 02/2024 by the Alliance Against Antisemitism in Healthcare of Australia	Since October 7, 2023: One-half reported feeling need to hide Jewish identity, and approximately two-thirds knew someone else who felt this need One-third reported experiencing antisemitism in the workplace Approximately two-thirds reported feeling unsafe or uncomfortable One-third reported avoiding group situations at work or social situations involving colleagues

**Medicine** Katz (2024)^26^ Pope (2024)^27^	Survey of 293 Jewish healthcare professionals in the UK, conducted in late 2023; findings reported in two media sources	95% reported an increase in antisemitism in their daily lives since October 7, 2023 73% reported experiencing at least one antisemitic incident; among these respondents, 70% reported that the source of at least one incident was within their own healthcare circles 44% had modified or were considering modifying their professional activities because of increased antisemitism Among respondents who had reported an antisemitic incident at work, only 15% believed that it has been managed appropriately

**Medicine** Starr (2024)^28^	Survey of 1,000 Canadian Jewish physicians, residents, and medical students, half from Ontario, conducted by the Jewish Medical Association of Ontario in December 2024; findings reported in a media source	31% reported considering leaving Canada because of increased antisemitism in medical institutions and schools 1% of respondents felt antisemitism was a severe problem in the medical field before October 7, 2023. This is vs 98% worried about impact of antisemitism on the healthcare system since the “Hamas-led 2023 pogrom in Israel’s south”^28^ 80% reported experiencing antisemitism in medical institutions since October 7, 2023 vs data before the terrorist attack: 29% had experienced prejudice in their medical community, 39% in hospitals, and 43% in academic settings 75% reported experiencing antisemitism in academic settings, and 64% reported experiencing it in hospitals since October 7, 2023 Respondents reported experiencing antisemitism through organization policies (57%), organizational communications (55%), personal interactions with colleagues (53%) and non-physician staff (41%), and through the administration (38%)

**Psychology** Brosbe et al. (2026)^29^	Survey of 218 Jewish mental health professionals in the US, conducted in 2025	91% regarded antisemitism as a problem, 93% believed it had increased during the previous years, and 74% reported personally experiencing antisemitism 39% reported antisemitic harassment Reported occupational effects of antisemitism at work included anxiety (74%), damaged relationships with colleagues (53%), and professional exclusion (21%) Qualitative responses described exclusion, erasure, and de-legitimization of aspects of Jewish identity and practice
**Healthcare Professions** Schwartz et al. (2025)^23^ Schwartz et al. (2026)^19^	Quantitative analysis of 3.43 million Twitter/X posts, 899,248 of which were from accounts of 224,405 self-identified healthcare professionals from 05/2023 to 03/2024	Posts related to antisemitism increased more than 5-fold during the study period Posts classified as promoting antisemitic stereotypes increased 2– to 4–fold Antisemitic conspiracy theories increased 1.9–fold, and Holocaust inversion increased 3.6–fold The authors concluded that “Social media, a powerful tool for extremist ideologies and spreading hate also enabled dramatic spread of antisemitism in healthcare”^23^

The initial PubMed search for antisemitism in the healthcare professions yielded 61 records. For context, parallel searches with the same search strings but instead using the terms *diversity*, *equity*, and *inclusion* (DEI), and *racism* yielded 732 and 3,824 records, respectively—12 and 63 times the number retrieved by the antisemitism search during the same period.

### Reported Examples of Antisemitism in the Health Professions Since October 7, 2023

This section summarizes incidents and experiences described in peer-reviewed publications, media reports, governmental documents, and organizational sources; the nature and evidentiary status of these sources vary.

#### Medicine

In the US, medical school faculty and health practitioners posted antisemitic tropes and derogatory comments about Jews in healthcare; medical students’ social media claimed Jews wielded disproportionate power (an antisemitic trope).^30^ Class content at the University of California–Los Angeles (UCLA) David Geffen School of Medicine (DGSM) labeled Jewish people as oppressors (demonization),^31^,^32^ and anti-Jewish hate graffiti appeared at University of California–San Francisco (UCSF).^6^ Jewish patients at UCSF hospitals feared revealing their identities, hospital staff shouted “intifada,” and social media of trainees and faculty rationalized terrorism against Jews.^6^,^33^–^35^ A UCSF physician was quoted as saying “When that chant goes up and is heard in the patients’ rooms, …. it’s a violation of our professional obligations as health care providers.”^34^

Protesters outside academic medical centers in New York City shouted “we don’t want no Zionists here” and carried signs including “Abolish Israel,” “Right of Return,” and “There is only one solution—intifada, revolution.”^36^ Jewish students and practitioners in health professions described isolation and fear of retribution for speaking out and for their safety.^2^ Jewish trainees scrubbed resumes and hid their religious beliefs.^37^,^38^ A leader of a Jewish organization at Brown University commented, in reference to a demonstration during a medical school commencement ceremony (which included chants heard from outside of “From the River to the Sea,” a slogan delegitimizing and calling for the elimination of the State of Israel), that “Jewish students are unfairly expected to bear the burden of unchecked micro and macro aggressions directed at them by peers.”^39^

At UCLA DGSM, Jewish students experienced “hostility and fear … from peers, colleagues, and administrators,” and “their concerns were not addressed by the university.”^6^ A UCSF Professor of Medicine was dismissed, but only after nearly two years of repeated anti-Zionist libels, unfounded accusations, and targeting of an alleged Israeli-born medical student.^6^ An Icahn Mount Sinai School of Medicine faculty member was terminated for supporting terrorism and anti-Israel libel.^40^

In addition to physicians’ social media vilifying Israel, some physicians at the Faculty of Medicine, University of British Columbia (UBC, Canada) “asserted that Jewish faculty should not be allowed to adjudicate resident matching because the examining doctors were Jewish and might be racist,” and in November, 2023, “1/3 of all UBC medical students signed a petition endorsing this call. Jewish learners who refused to sign were harassed by staff and students.”^25^ Despite almost 300 Jewish physicians signing a letter expressing concern about tolerance of Jew hatred and hostile academic environment, antisemitism was not recognized by leadership.^25^ A valedictory speech for the 2024 class of the Max Rady College of Medicine, University of Manitoba included demonization of Israel, and the student who delivered it received a standing ovation from fellow graduating students.^41^

A UK tribunal suspended a physician for 15 months “after ruling her antisemitic and extremist social media posts fell far below professional standards and posed a high risk of re-offending.”^42^,^43^ But a medical journal termed these posts “allegations” of antisemitic remarks.^43^ The physician’s social media activity included three reported examples: (1) describing the temporary restriction of emergency department access in Manchester, which was intended to prioritize victims of a local synagogue attack, as evidence of “exceptionalism for Jews;” (2) sharing a social media post that characterized media coverage of the attack as “Jewish supremacism;” and (3) calling on Jews to denounce Israel or renounce Israeli citizenship. This physician was subsequently arrested after violating bail conditions and was charged with inviting support for Hamas.^44^

In a separate case in Belgium, a radiologist reportedly identified a patient as Jewish in the patient’s medical documentation, raising concerns about privacy and professional conduct. The physician was later suspended after antisemitic cartoons posted on his social-media account in 2023 came to light.^45^

#### Dentistry

Reports have described antisemitism in dental workplaces, academic institutions, and international dental organizations, including harassment and hostility directed at individuals because of their Jewish identity or support for Israel.^46^ “Some Jewish professionals are now hesitant to wear faith symbols or identity in their own workplaces, fearing retaliation, ostracism, or subtle professional retribution” and “quiet but persistent normalization of exclusion which occurs often under the radar but is just as damaging.”^46^ Dentists in Miami and Boston tore down posters of Israelis kidnapped by Hamas terrorists and were ultimately fired.^47^,^48^ A US congressman called for revoking the license of a Florida dentist who made antisemitic statements when preaching in his role as an imam after the October 7, 2023 terrorist attack by Hamas against Israel.^49^

#### Nursing

An Australian nurse and lifestyle influencer posted a video in which she appeared to perform a Nazi salute, then issued an apology.^50^ Australian nurses threatened Israeli patients during an online chat. One boasted she would refuse to treat Israeli patients and threatened to kill them. Another claimed he had sent Israeli patients to “Jahannam” (Arabic for hell).^51^ Both were dismissed from their positions and criminally charged, with their cases scheduled for trial in August 2026. An Oregon Health & Science University nurse was terminated for referring to advocates for Israel as “vermin,” writing she would “… not treat them.”^52^

#### Psychology

An open letter to the APA from Psychologists Against Antisemitism and others (4,000 signatories) documented the following: (1) “insensitivity toward Jews, a lack of concern regarding antisemitism, minimization of aggression towards the Jewish people, and outright hostility and prejudice toward Jews and Jewish heritage,” including “listservs containing antisemitic discourse, often masked as anti-Zionism;”^53^ and (2) the APA offering educational credits for members to attend conferences where speakers endorsed violence against Jews and Israelis, invoked antisemitic tropes, distorted the Holocaust, minimized Jewish experiences, and pathologized the Jewish people’s connection to their indigenous homeland.^53^

An article in the APA’s flagship journal *American Psychologist* showed “clear animus to Jewish people and Israel,” repeatedly labeled Israel a “settler-colonial” and “apartheid” state, distorted history, and advocated for “decolonial psychology practice,” which “politicizes the field of scientific psychology and falls drastically short of scholarly standards.”^54^ A psychologist who directs Villanova University’s Counseling Center associated Zionism with fascism and colonialism during a professional psychology conference in Philadelphia.^55^

The Brandeis Center and Physicians Against Antisemitism criticized Decolonizing Therapy for identifying Zionism as a root cause of mental illness.^56^ Marcus (of the Brandeis Center) argued that this practice equates Zionism—described as “an integral component of Jewish identity for many Jewish Americans” and as the belief that Jews have a right to self-determination in Israel, their ancestral homeland—with colonialism and oppression.^57^ Marcus elaborated that this “xenophobic hatred under the guise of therapy” delegitimizes Jewish historical, cultural, and ethnic connections to Israel and denies the Jewish people’s more than 3,000-year history with the land.^57^ Decolonial and liberation therapies incorporate antisemitic narratives including anti-Zionism and identity-based discrimination into cognitive behavioral therapy (CBT).^58^ Allowing such narratives “to shape theory and practice undermines the profession scientifically, ethically, and clinically.”^58^

The lack of meaningful response to these developments prompted a US congressional investigation,^59^ which one commentary described as evidence that the “APA’s antisemitism reckoning has finally arrived.”^60^ The state of Illinois formally reprimanded a therapist who had created a list of “Zionist” therapists and encouraged colleagues not to refer clients to those therapists.^61^

#### Social work

Responses to antisemitism within the social work profession have lacked action steps, such as encouraging social workers “to reflect on their own internalized anti-Semitic biases.”^62^ A survey of US social workers’ experiences with antisemitism in both education and the workplace revealed antisemitic comments from peers, office workers, and staff and identified the following themes: (1) being considered “white and privileged” to minimize and dismiss Jewish experiences, (2) “micro-aggressions” against Jews seldom addressed and often dismissed, and (3) merging antisemitism with anti-Zionism and the demonization of Israel, most prevalent in graduate schools.^63^ These researchers noted that antisemitism was seldom included in social work education or diversity and inclusion programs, inconsistent with its approach to social justice and maintaining a truly inclusive profession.^63^ Other researchers likewise noted its omission from institutional responses since October 7, 2023.^64^ This omission is inconsistent with the profession’s commitment to social justice and to maintaining a truly inclusive profession.^62^ They thus recommend including the topic of antisemitism in social work curricula and diversity and inclusion programs in accordance with the profession’s ethics and competencies through the Council on Social Work Education, the accrediting agency for social work education in the US and its territories. In 2024, an Ontario-based Jewish social worker was publicly labeled “racist’” and “anti-Arab” for challenging her colleague’s assertion that Zionism is a “genocidal ideology.”^65^

The Jewish Social Work Consortium report described the targeting of Jewish social workers on national listservs and antisemitic rhetoric in academic settings, with Jewish students studying social work termed “racist” and “white supremacists.”^66^ The report also raised serious concerns about the National Association of Social Workers, the largest membership organization of professional social workers (110,000 members), including its two-month delay in publicly addressing the massacre of October 7, 2023 and its allegedly inadequate response to antisemitism and inadequate efforts to counter it in the aftermath. Columbia University reached a settlement in a former School of Social Work student’s Title VI lawsuit alleging antisemitic discrimination.^67^

#### Occupational therapy

It has been deemed essential within the occupational therapy field for faculty to develop and implement programs for practitioners, students, and other professionals and create a culture of belonging in light of the sharp rise in antisemitism “to prevent occupational injustice among Jewish students.”^68^

In summary, the themes or patterns across these specific examples of antisemitism in the health professions are similar, if not identical. They encompass manifestations of antisemitism as outlined in the IHRA guidelines, ranging from outright threats of bodily harm against Jews to the use of antisemitic tropes and libels to demonize and delegitimize Israel and apply double standards to it—Israel being the ancestral homeland of the Jewish people and an integral component of the identity of most Jews.

### Actionable Recommendations for Countering Antisemitism in the Health Professions

Countering antisemitism in medicine is a “moral imperative,”^2^ where the “4Es” serve as an action framework,^2^ i.e. Education, Engagement, Empathy, and Enforcement. These 4Es were recently endorsed by the Antisemitism Policy Trust of the UK in their Antisemitism in the Healthcare Sector Since October 7, 2023 report.^69^ A recent study found that people holding antisemitic attitudes and tropes were less educated on Jewish history,^70^ highlighting the importance of education in reducing antisemitic beliefs.^15^ Education in this framework includes history of medicine’s complicity in the Holocaust,^2^,^71^ with an important caveat on any Education component in this domain: “Honoring the memory of the Holocaust is a sacred obligation. Yet it cannot be done effectively or have any real meaning in a context divorced from the current struggle for Jewish survival against a rising tide of bigotry, hatred, and violence.”^72^ In other words, “Don’t mourn the Holocaust while supporting the genocide of living Jews.”^72^ Some reported patterns—particularly the dehumanization, exclusion, and professional targeting of Jewish healthcare workers—echo patterns documented in pre-Holocaust Nazi Germany. One contemporary example of such echoes in medicine is the Committee of Interns and Residents, the largest union of resident and fellow physicians in the US, representing more than 37,000 physicians, which has supported figures associated with anti-Israel terrorist organizations, recommended the exclusion of Israeli employees from hospitals, adopted resolutions with libelous claims of “apartheid” and “genocide” against Israel, supported the Boycott, Divestment, and Sanctions (BDS) movement, and required dues from federally funded trainees—actions that help perpetuate antisemitic bias in medicine.^73^,^34^ The Committee of Interns and Residents is currently the subject of a US Congressional investigation.^73^,^34^ This history underscores the need to recognize and address the broader implications of the dehumanization, exclusion, and persecution of Jewish health professionals, as well as the extent to which antisemitic hatred can escalate.^2^,^71^ It also demonstrates how antisemitic incitement can lead—and historically has led—to deadly violence. Actionable recommendations and actions taken based upon the narrative review are presented in Table 5.

**Table 5:** Actionable Recommendations and Examples of Actions Taken.

Citations	Actionable Recommendation and Action Taken
Wald and Roth (2024)^2^ Getzoff Testa et al. (2024)^75^	**4Es:2 (1) Education:** (**a)** promoting better understanding of Judaism, the Jewish people, Jewish identity, and Jewish experience in clinical practice, research, and training
Czech et al. (2023)^76^ Chelouche et al. (2023)^77^ Wald and Roth (2024)^2^ Reis and Wald (2024)^78^ Chelouche (2025)^79^ Adler et al. (2025)^80^ Wald and Ehrenfeld (2025)^81^ Adler and Grant-Kels (2024)^71^ Cox and Marlowe (2024)^63^	**4Es:2 (1) Education:** (**b)** required teaching of history of medicine’s complicity in the Holocaust and contemporary relevance for all humanity “to understand the dangerous potential of antisemitism”^90^ and for shaping moral Professional Identity Formation; **(c)** proactive education on anti-Zionist libel, hate, and stigma
Wald and Roth (2024)^2^	**4Es: (2) Engagement, (3) Empathy, and (4) Enforcement of policies** including applying anti-discrimination standards consistently
Nazarian (2023)^82^ Beck et al. (2025)^83^ Getzoff Testa et al. (2024)^75^ Ferreira et al. (2024)^84^ Ancis and Weisskirch (2025)^85^ Walker et al. (2025)^86^	**(5) Revamping Diversity, Inclusion, and Anti-Bias Training Initiatives to include Judaism, Jewish Identity, and Antisemitism.** Resisting “DEI fueled politicization”^82^ Social Work: Use NASW Code of Ethics to address antisemitism Psychology: “Roadmap”—inclusion of antisemitism in APA Office of Equity, Diversity, and Inclusion
Katz (2024)^26^ Nystrom (2025)^87^ Jewish Social Work Consortium report included in Kogan (2025)^88^ Strous (2025)^89^	**(6) Professional and Legal Associations:** implement educational and policy enforcement initiatives, promote culturally safe care for Jewish patients; push back against weaponization of healthcare against Israel
Joint Statement of the ADL, AEN, and Psychologists Against Antisemitism included in ADL (2025)^54^ National Association of Social Workers (2026)^90^	**(7) Association and Inter-Association Action:** July 2025: Joint statement (ADL, AEN, Psychologists Against Antisemitism): against an *American Psychologist* article showing “clear animus” to Jewish people and Israel. A previous joint statement (ADL/AEN) in May 2025 urged the APA to address rising tide of antisemitism including anti-Israel bias January 2026: The NASW opposing the International Federation of Social Workers’ motion (ultimately defeated) that issued a formal “censure” against the Israeli Union of Social Workers
US House Committee on Energy and Commerce (2024)^35^ Trovato (2025)^31^ Konecky (2025)^91^ Whyte (2025)^7^ Song (2025)^39^ Heller (2025)^92^ Dupre (2025)^93^ Department of Health and Social Care, The Rt Hon Sir Keir Starmer KCB KC MP and The Rt Hon Wes Streeting MP (2025)^94^ Dyer and Iacobucci (2025)^43^ Bilger (2026)^95^ Heller (2025)^42^	**(8) Governmental Interventions:** February 2025: US Department HHS civil rights investigation over reported antisemitism at Harvard Medical School, Columbia University Vagelos College of Physicians and Surgeons, Warren Alpert Medical School of Brown University, and Johns Hopkins University School of Medicine August 2025: US Congress Committee on E&W investigation of antisemitism at UCLA, UCSF, and University of Illinois medical schools October 2025: UK government urgent action to tackle antisemitism within education and National Health Service December 2025: US House Committee on E&W investigates antisemitism within the APA February 2026: US Justice Department’s Civil Rights Division and US HHS investigates Lincoln Memorial University–DeBusk College of Osteopathic Medicine regarding discrimination against Jewish students
Hart and Garlick (2025)^96^ Frenk (2025)^97^ Harvard University (2025)^98^ Jewish Federations of North America (2024)^99^	**(9) On Campus Action** Formation of Jewish ERGs, e.g. Mass General-Brigham, Columbia, Sick Kids Toronto Congressional advocacy to prevent denying Jewish employees at medical institutions the opportunity to form ERGs UCLA Initiative to Combat Antisemitism Harvard University Task Force on Combating Antisemitism and Anti-Israel Bias
Adler and Grant-Kels (2024)^100^ Edelson (2024)^101^ Reis and Wald (2024)^78^ Roth et al. (2024)^102^ Roth and Wald (2025)^103^ Walker et al. (2025)^86^ Schwartz et al. (2026)^19^ Strous (2025)^89^	**(10)** Publications and correspondence to rebut non-evidence-based allegations and omissions grounded in anti-Israel bias in medical/scientific journals and medical society and medical union statements that can fuel antisemitic sentiment
Change.org (2023)^73^ Deutch (2025)^104^ Deutch (2025)^105^ Heller (2025)^106^ Heller (2025)^107^ Wald et al. (2025)^108^ Hauser (2025)^109^ Agronin (2026)^74^	**(11) Advocacy Letters, Testimony, and Related Initiatives** Letter against the Committee of Interns and Residents (US doctors’ union) about perpetuating antisemitic bias Letter against American Academy of Pediatrics leadership advocating for a doctor identified as a Hamas terrorist organization member “Open Letter on Antizionism and the Weaponization of Medicine: A Call from Jewish and Allied Health Professionals” from >1,000 North American Jewish health professionals providing evidence of “near-constant abuse” and a “far longer erosion of professional norms”; identified and emphasized the “anti-Zionist problem in medicine”; motion brought by the Jewish Medical Association of the UK president after the British Medical Association passed three anti-Israel resolutions at its annual conference Letter asking Royal Children’s Hospital in Melbourne to cancel a program on “Children and War” with panelists that had engaged in “… hateful, partisan campaigns against Israel and its supporters” Letter calling for (a) health professionals and their professional associations’ condemnation of flagrant international law violations of hostage taking and torture and murder of prisoners by Hamas (October 7, 2023 invasion and murderous rampage) and condemning any attempts to justify or rationalize these acts; (b) learning from history of medicine’s complicity in Nazism and the Holocaust and its contemporary relevance including fighting antisemitism and all hate; and (c) medical journals to uphold publication standards on this conflict with documentable facts, not unfounded accusations or deliberate omissions MIT chapter of Kalaniyot founded in the aftermath of October 7, 2023 with aims of advancing academic excellence by deepening ties with Israeli researchers and building a supportive and inclusive campus community Written testimony before the Subcommittee on Health, Employment, Labor, and Pensions Committee on Education and Workforce United States House of Representatives Hearing on “Bad medicine: politics, unions, and antisemitism in health care”

Sources include peer-reviewed articles, commentaries, organizational statements, governmental documents, media reports, and advocacy materials; these source types do not carry equivalent evidentiary weight.

4 Es, Education, Engagement, Empathy, and Enforcement; ADL, Anti-Defamation League; AEN, Academic Engagement Network; APA, American Psychological Association; DEI, diversity, equity, and inclusion; E&W, Education and the Workforce (US House Committee); ERGs, Employee Resource Groups; HHS, US Department of Health and Human Services; MIT, Massachusetts Institute of Technology; NASW, National Association of Social Workers; UCLA, University of California–Los Angeles; UCSF, University of California, San Francisco.

## DISCUSSION

Our narrative review identified widespread reports of antisemitism in health professions education and practice across several countries, with multiple studies and surveys documenting significant increases after the Hamas terrorist attack of October 7, 2023, the largest massacre of Jewish civilians in a single day since the Holocaust. The Holocaust researcher Yehuda Bauer pointed out that antisemitism endangers all humanity.^110^ Hence, by interpretation, it is not only a danger to all Jewish people, but to all people worldwide.

While HPE and practice have identified and mitigated injustices because all bigotry, hatred, and discrimination cannot be tolerated, antisemitism as a form of bigotry, hatred, and discrimination has not been adequately addressed. This includes its absence from diversity and inclusion initiatives in medical and mental health professions training and practice.^2^,^63^ The relative paucity of literature on antisemitism in healthcare raises the question of whether we may be encountering yet another double standard manifestation of antisemitism, i.e. creating and enabling a hostile climate leading to fears of retribution for speaking up in publication format. Respondents in multiple surveys reported lack of support or mixed experiences from their institutions and lack of organizational support.^15^,^23^

Targeted review of references emerging from PubMed and ProQuest sources with rigorous application of search terms identifying antisemitism in healthcare education and practice reduced the number of extracted references. We, however, obtained a relatively robust number of relevant citations through “hand search” of citations within the extracted references from both PubMed and ProQuest sources. We noted that a considerable number of these had not been detected by PubMed or ProQuest due to their being books, government statistics, and database URLs, as well as inclusion of social work and psychology journals whose coverage in PubMed is limited. While our review described quantitative and qualitative data on the scourge of antisemitism in the health professions, what did not emerge is also significant. Aside from one letter to the editor^81^ and one personal essay of experienced antisemitism,^38^ there were no citations of medical education journals addressing this scourge in medical education journals (e.g. *Academic Medicine*, *Medical Education*, *Medical Teacher*), and no substantial body of empirical research or coordinated initiatives from medical associations or accrediting bodies emerged in the searches. Possible explanations include the recency of the phenomenon, the scope and limitations of the searches, publication practices, and reluctance to address a contentious subject; this narrative review cannot specify the reasons for the limited literature. Nevertheless, the relative absence of educational sources about Judaism, Jewish identity, and antisemitism within diversity and inclusion initiatives is notable. Anecdotally, the degree of manuscript rejections in this domain is problematic and the subject of a future analysis. While some publications on this issue were extracted for the mental health professions, these articles raised serious concerns about lack of appropriate action by professional organizations. Concerns in general regarding inadequate addressing of the antisemitism “virus” and its “mutation”^3^ persist in the health professions. These concerns persist even with governmental investigations into antisemitism in medical schools and into a professional psychology organization (US), as well as into the UK’s National Health Service.^42^

Qualitative reports documented antisemitism in healthcare impacting trainees and professionals with serious concerns for Jewish patients. Results were consistent with a “mutation” of contemporary antisemitism,^3^ or “transmutation,”^111^ with Jew hatred and bigotry manifesting substantially in anti-Israel libel and anti-Zionist forms.^66^ This manifests as demonization and delegitimization, and/or double standards applied to Israel^111^,^112^ within social media, commencement ceremonies, classes, panels, medical journal publications, and ostracizing and erasure of Jewish experience. Whether “antizionism” embraced in “dangerous ignorance”^113^ is a form of antisemitism^19^ “incontestably” so,^114^ or a distinct contemporary form of anti-Jewish hostility and hate, all forms of Jew hatred with “Othering” of Jews^113^ are unacceptable, inconsistent with professionalism, and deserve urgent action to prevent civil rights violations and negative impact on patient care. According to the ADL, “accompanying the IHRA definition are 11 examples that ‘may serve as illustrations’ of how antisemitism manifests contemporaneously, ranging from age-old anti-Jewish tropes, to Holocaust denial, to certain expressions of animus toward the Jewish State of Israel that may cross the line into antisemitism.”^115^ A recent exemplar is bias in a medical journal publication in the form of antisemitic Holocaust inversion, assertion that the “Holocaust damaged Jewish national identity,” use of trope-echoing antisemitic libels, as well as demonizing and delegitimizing Israel.^89^,^116^,^117^ Additional exemplars within medical journals more recently include demonizing Israel concerning the Hezbollah-terrorist group-sustained war in Lebanon,^118^ decontextualizing war (and resulting healthcare issues) from terrorist antecedents,^119^ and “selective mutism” in regard to assaults on hospitals and doctors by pro-regime Iranian forces.^120^

Particularly striking are the significant issues in psychology and social work with the irony of pervasive antisemitism in social work, e.g. noted by the Jewish Social Work Consortium as bias contradicting the profession’s ethical code that emphasizes “… social justice, dignity, worth of the person, and the significance of human relationships,” and “social justice” distorted to include “harmful stereotyping of Jewish and Israeli individuals”^88^ with increasing anti-Israel bias. Antisemitic prejudice under the guise of anti-Zionism or anti-Israel bias is not legitimate nor morally righteous. The joint statement of ADL, the Academic Engagement Network, and Psychologists Against Antisemitism captured it well—”A professional environment free from bias and bigotry of all forms including antisemitism” is needed.^54^

Moral responsibility in the health professions includes action when necessary, such as published replies to counter politicized agendas, contemporary libel, and one-sided perspectives appearing in medical journals, which has had impact.^19^,^89^ This includes a publication on unprofessional antisemitic displays at US medical school commencements picked up in major media outlets and cited in federal investigations of medical schools and healthcare institutions including Harvard Medical School, Alpert Medical School of Brown University, Columbia University Vagelos College of Physicians and Surgeons, Univer-sity of California, San Diego School of Medicine, UCSF, UCLA DGSM, Stanford University School of Medicine, and University of Illinois College of Medicine.^24^,^121^ Other examples include a journal theme issue addressing antisemitism and social work,^64^ and social workers’ organized opposition to an antisemitic motion brought by the International Federation of Social Workers leading to this motion being ultimately rejected.^122^

## LIMITATIONS

This narrative review has several limitations. The searches were restricted to English-language sources published after October 7, 2023, and the ProQuest search focused on medicine, psychology, and social work. The included evidence was heterogeneous and comprised peer-reviewed studies, media reports, governmental and organizational documents, commentaries, and advocacy materials. Publication, reporting, and selection bias may have influenced the evidence identified. Differences in study design, sampling, and source type should therefore be considered when interpreting the findings. Nevertheless, the reported patterns and available evidence provide an indication of the current prevalence of antisemitism within the populations and professional settings studied.

## CONCLUSION

This narrative review characterized the extent of antisemitism in health professions education and practice internationally and its dramatic increase since the Hamas massacre of October 7, 2023 in Israel. As such, it illustrates the moral imperative for education, engagement (for critical reflection), empathy, and enforcement,^2^ with educational topics of Judaism, Jewish identity, history of health professions’ complicity in the Holocaust, countering Holocaust inversion, and on origins and persistence of antisemitism. Mandatory inclusion in “diversity and inclusion” and/or cultural humility curricula as well as research on best practices and assessment of outcomes is called for in order to properly address and counter this scourge. Antisemitism is antithetical to and violates ethical oaths and charters of the health professions charged with assuring that clinical and learning environments are free of bias and hate.^85^ It also highlights the need for editorial vigilance to maintain journalistic integrity in medical/scientific journals to counter antisemitic anti-Israel bias which has appeared and can fuel antisemitic sentiment and actions. We must take firm action to counter all of this more broadly as a “malignant normality” which manifested in the ethical perversion and complicity of physicians in Nazism and the Holocaust.^123^ Today, antisemitic prejudice and associated forms of anti-Israel bias and anti-Zionism must be countered. It is our duty and moral responsibility within clinical care, research, teaching, systems policy, and administrative leadership in the health professions.

## Supplementary Information



## Abbreviations

ADL: Anti-Defamation League
APA: American Psychological Association
DGSM: David Geffen School of Medicine
HPE: Health Professions Education
IHRA: International Holocaust Remembrance Alliance
PRISMA: Preferred Reporting Items for Systematic Reviews and Meta-Analyses
UBC: University of British Columbia
UCLA: University of California, Los Angeles
UCSF: University of California, San Francisco
UK: United Kingdom
US: United States
