# Supplementary material for: Anatomy of Antisemitism in Health Professions Education and Practice: A Narrative Review
Source: Rambam Maimonides Med J. 2026 Jul 31;17(3):e0021. doi: 10.5041/RMMJ.10581 (PMC13436919; doi:10.5041/RMMJ.10581)
Supplement: Supplementary file 1 [file rmmj-17-13-e0021_supplement.docx]

This appendix has been provided by the authors for the benefit of readers

Supplement to Anatomy of Antisemitism in Health Professions Education and Practice: A Narrative Review

Wald HS, Roth S. Anatomy of Antisemitism in Health Professions Education and Practice: A Narrative Review. Rambam Maimonides Med J 2026;17 (3):e0021. Review. doi:10.5041/RMMJ.10581

# Sources Included in the Narrative Review

## Supplement A: PubMed Records Included in the Review (October 7, 2023–March 2026), *n*=23.

This list represents the records shown in Figure 1 and in the text that were detected using the PubMed search.

1. Adler NR, Grant-Kels JM. Antisemitism and dermatology in Nazi Germany and beyond. Clin Dermatol 2024;42:508–12. <https://doi.org/10.1016/j.clindermatol.2024.07.001>
2. Adler NR, Grant-Kels JM. Hatred has no place in medicine: the dermatologist’s role in countering antisemitism and all other forms of racism. Clin Dermatol 2024;42:91–2. <https://doi.org/10.1016/j.clindermatol.2023.11.007>
3. Adler NR, Rakov JA, Adler KA, Grant-Kels JM. A call for moral clarity in the fight against antisemitism. Lancet 2025;405:466–7. <https://doi.org/10.1016/s0140-6736(24)00150-8>
4. Adler NR, Rakov JA, Adler KA, Grant-Kels JM. All children pay the price for antisemitic bias. Lancet 2024;403:143–4. <https://doi.org/10.1016/s0140-6736(23)02636-3>
5. Carey LB, Cohen J, Gabbay E, et al. Research reviews, Israel, antisemitism and scales for religious and spiritual assessment. J Relig Health 2026;65(1):1–5. doi: 10.1007/s10943-026-02573-x.
6. Chelouche T. Post-October 7th in the academy: legacies of Nazism and the resurgence of antisemitism in contemporary university education. Isr Med Assoc J 2025;27:547–51. PMID: [40932204](https://pubmed.ncbi.nlm.nih.gov/40932204/)
7. Correction to “The American Psychological Association and antisemitism: toward equity, diversity, and inclusion” by Walker et al. (2024). Am Psychol 2025;80:119. [https://doi.org/10.1037/‌amp0001396](https://doi.org/10.1037/amp0001396)
8. Dyer C, Iacobucci G. Antisemitism in medicine: as Wes Streeting vows sweeping changes, how did we get here? BMJ 2025;391:r2140. <https://doi.org/10.1136/bmj.r2140>
9. Farber NB, Fram MS. The danger of ideology: social work, Israel, and anti-semitism. Soc Work 2024;69:204–6. <https://doi.org/10.1093/sw/swad052>
10. Ferreira AL, Sow YN, Grant-Kels JM. Dermatology’s ethical obligations to diversity, equity, and inclusion and antisemitism. Clin Dermatol 2024;42:721–2. <https://doi.org/10.1016/j.clindermatol.2024.06.005>
11. Fishman AC, Auerbach C. Antisemitism in American healthcare: the role of workplace environment. J Relig Health 2026;65:502–13. <https://doi.org/10.1007/s10943-025-02330-6>
12. Gordon M, Teitel J, Rosenberg T, Oratz R, Katz N, Katz D. Antisemitism in medicine: an international perspective. Rambam Maimonides Med J 2025;16:e0004. <https://doi.org/10.5041/rmmj.10536>
13. Michelson KN, Fishman AC, Feinberg EC, et al. Antisemitism in American healthcare: a survey study of reported experiences. J Gen Intern Med 2025;40:1940–3. <https://doi.org/10.1007/s11606-024-09159-x>
14. National Association of Social Workers. NASW’s Response to journal commentary on anti-semitism in social work. Soc Work 2024 Feb 1:swae010. <https://doi.org/10.1093/sw/swae010>
15. Reis SP, Wald HS. The Hamas massacre of Oct 7, 2023, and its aftermath, medical crimes, and the Lancet commission report on medicine, Nazism, and the Holocaust. Isr J Health Policy Res 2024;13:19. <https://doi.org/10.1186/s13584-024-00608-w>
16. Roth S, Wald HS. Reply to Babar and to Abu Fraiha and Leibowitz — No place for unprofessionalism including offensive antisemitic symbols and regalia at medical school commencement ceremonies. Rambam Maimonides Med J 2025;16:e0012. <https://doi.org/10.5041/rmmj.10547>
17. Roth S, Wald HS. US medical schools’ 2024 commencements and antisemitism: addressing unprofessional behavior. Rambam Maimonides Med J 2025;16:e0001. <https://doi.org/10.5041/rmmj.10537>
18. Schwartz DM, Leiba R, Feldman CL, et al. Misleading allegations and failure to recognize the shifting faces of antisemitism: a response to J.J. Amon. J Relig Health 2026;65:480–501. <https://doi.org/10.1007/s10943-025-02492-3>
19. Schwartz DM, Leiba R, Feldman CL, et al. Social media, survey, and medical literature data reveal escalating antisemitism within the United States healthcare community. J Relig Health 2025;64:206–23. <https://doi.org/10.1007/s10943-024-02191-5>
20. Van As AB, Sidler D, Allema JH, et al. Response: why solidarity with all children suffering in wars is not antisemitic. S Afr Med J 2025;115. Available at: <https://hdl.handle.net/10520/ejc-m_samj_v115_n5_a5> (accessed April 16, 2026).
21. Wald HS, Roth S. The moral imperative of countering antisemitism in US medicine - a way forward. Am J Med 2024;137:915–17. <https://doi.org/10.1016/j.amjmed.2024.06.015>
22. Walker LE, Cole E, Friedman SL, Rom-Rymer B, Steinberg A, Warshaw S. Scholarship, not politics: reply to Eidelson (2025). Am Psychologist 2025;80:122–3. <https://doi.org/10.1037/amp0001483>
23. Walker LEA, Cole E, Friedman SL, Bom-Rymer B, Steinberg A. The American Psychological Association and antisemitism: toward equity, diversity, and inclusion. Am Psychol 2025;80:106–19. <https://doi.org/10.1037/amp0001369>

## Supplement B: ProQuest Records Included in the Review (October 7, 2023–March 2026), *n*=46.

These records represent those shown in Figure 2 and in the text that were detected using the ProQuest search.

1. Ahmed, L. Jewish nurse sues NYU Langone, claiming revoked bonus over pro-Israel posts. Washington Square News 2025; September 8. Available at: <https://nyunews.com/news/2025/09/08/langone-nurse-sued-antisemitism-instagram/> (accessed April 16, 2026).
2. Anti-Defamation League and Academic Engagement Network. ADL and AEN urge the American Psychological Association to address antisemitism. ADL and AEN 2025; May 30. Available at: <https://academicengagement.org/wp-content/uploads/2025/05/APA-Statement-FINAL_5.30.2025.pdf> (accessed April 16, 2026).
3. Armanini, K. University of Illinois College of Medicine faces congressional antisemitism investigation. Chicago Tribune 2025; August 26. Available at: <https://www.chicagotribune.com/2025/08/26/university-of-illinois-antisemitism-investigation/> (accessed April 16, 2026).
4. Australian Associated Press. Bankstown nurse sacked over viral video has charge of threatening to kill Israeli patients dropped. The Guardian 2025; September 16. Available at: <https://www.theguardian.com/australia-news/2025/sep/16/bankstown-nurse-sacked-over-viral-video-has-charge-of-threatening-to-kill-israeli-patients-dropped-ntwnfb> (accessed April 16, 2026).
5. Ceja A. Congress investigates claims of antisemitism at UIC College of Medicine. The Daily Illini 2025; September 2. Available at: <https://dailyillini.com/news-stories/university-news/2025/09/02/congress-investigates-antisemitism/> (accessed April 16, 2026).
6. Dupré B. UIC medical school under congressional scrutiny in antisemitism probe. Crain’s Chicago Business 2025; August 25. Available at: <https://www.chicagobusiness.com/education/uics-college-medicine-faces-federal-antisemitism-probe/> (accessed April 16, 2026).
7. Editorial Staff. Editorial: APA’s Antisemitism reckoning has finally arrived. Philadelphia Jewish Exponent 2025; December 23. Available at: <https://www.jewishexponent.com/editorial-apas-antisemitism-reckoning-has-finally-arrived/> (accessed April 16, 2026).
8. Greenberg Z. Villanova faces blowback after director of counseling center associates Zionism with fascism in presentation. The Philadelphia Inquirer 2025; December 14. Available at: <https://www.inquirer.com/education/nathalie-edmond-villanova-counseling-psychology-conference-youtube-20241214.html> (accessed April 16, 2026).
9. Hauser B. Inside a new MIT initiative that aims to offer solution to what it sees as widespread bias against Israeli scientists. Boston Globe 2025; August 4. Available at: https://www.bostonglobe.com/2025/08/04/metro/mit-kalaniyot/ (accessed April 16, 2026).
10. Heller M. ‘Decolonizing therapy’ and the erasure of Jewish identity in mental health. The Jerusalem Post 2025; July 29. Available at: <https://www.jpost.com/diaspora/antisemitism/article-862643> (accessed April 16 2026).
11. Heller M. ‘Jews are white’: U.S. minority psychologists’ coalition rejects Jewish ethnic recognition. The Jerusalem Post 2026; February 16. Available at: <https://www.jpost.com/diaspora/antisemitism/article-886837> (accessed April 16, 2026).
12. Heller M. All three anti-Israel motions at British Medical conference pass with majority vote. The Jerusalem Post 2025; June 25. Available at: <https://www.jpost.com/diaspora/antisemitism/article-859006> (accessed April 16, 2026).
13. Heller M. Antisemitic UK doctor Rahmeh Aladwan suspended for 15 months, blames ‘Jewish lobby.’ The Jerusalem Post 2025; November 30. Available at: <https://www.jpost.com/diaspora/antisemitism/article-876717> (accessed April 16, 2026).
14. Heller M. Axed children’s hospital event would have endangered Jewish staff, Australian doctor tells ‘Post.’ The Jerusalem Post 2025; September 15. Available at: <https://www.jpost.com/diaspora/antisemitism/article-867545> (accessed April 16, 2026).
15. Heller M. Belgian hospital suspends radiologist pending investigation into antisemitic social media posts. The Jerusalem Post 2025; September 3. Available at: <https://www.jpost.com/diaspora/antisemitism/article-866236> (accessed April 16, 2026).
16. Heller M. Perth doctor harassing Zionist medics, sources tell ‘Post’ - exclusive. The Jerusalem Post 2025; October 9. Available at: <https://www.jpost.com/diaspora/antisemitism/article-869926> (accessed April 16, 2026).
17. Heller M. Rabid pro-Hamas, antisemitic NHS doctor referred to tribunal, may lose license. The Jerusalem Post 2025; September 3. Available at: <https://www.jpost.com/diaspora/antisemitism/article-866250> (accessed April 16, 2026).
18. Heller M. UK gov. ministers set to launch antisemitism crackdown. The Jerusalem Post 2025; October 12. Available at: <https://www.jpost.com/diaspora/antisemitism/article-870240> (accessed April 16, 2026).
19. Heller M. US House launches probe into antisemitism allegations at American Psychological Association. The Jerusalem Post 2025, December 14. Available at: <https://www.jpost.com/diaspora/antisemitism/article-880309> (accessed July 16, 2026).
20. House Committee on Energy & Commerce. E&C, E&W Republicans demand answers from Columbia University. Congressional Press Release 2024; September 19. Available at: <https://energycommerce.house.gov/posts/e-and-c-e-and-w-republicans-demand-answers-from-columbia-university> (accessed April 16, 2026).
21. Jerusalem Post Staff. Sydney hospital scrubs pro-Palestine social media posts after nurses caught in antisemitism scandal. The Jerusalem Post 2025; February 12. Available at: <https://www.jpost.com/diaspora/antisemitism/article-841797> (accessed April 16, 2026).
22. Kaleem J. GOP widens UC antisemitism investigations, hitting UCLA, UC San Francisco medical schools. Los Angeles Times 2025; August 25. Available at: <https://www.latimes.com/california/story/2025-08-25/ucla-ucsf-medical-school-house-education-committee> (accessed April 16, 2026).
23. Knight H. In San Francisco, doctors feud over ‘Do No Harm’ when it comes to war protests. New York Times 2024; June 24. Available at: <https://www.nytimes.com/2024/06/24/us/israel-hamas-war-sf-doctors.html> (accessed April 16, 2026).
24. Knoblauch N. University settles in former School of Social Work student’s Title VI lawsuit alleging antisemitic discrimination. Columbia Spectator 2025; June 24. Available at: <https://www.columbiaspectator.com/news/2025/06/23/university-settles-in-former-school-of-social-work-students-title-vi-lawsuit-alleging-antisemitic-discrimination/> (accessed April 16, 2026).
25. Konecky M. David Geffen School of Medicine under investigation for antisemitism claims. Daily Bruin 2025; August 25. Available at: <https://dailybruin.com/2025/08/25/david-geffen-school-of-medicine-under-investigation-for-antisemitism-claims> (accessed April 16, 2026).
26. Lieberman J. From neighbors and friends, why betrayal still haunts Jews to this day – opinion. The Jerusalem Post 2025; August 23. Available at: <https://www.jpost.com/opinion/article-864965> (accessed April 16, 2026).
27. Marcus KL. America’s mental health field is overrun with antisemitism. It’s dangerous (USA Today). Brandeis Center website. 2025; July 17. Available at: <https://brandeiscenter.com/americas-mental-health-field-is-overrun-with-antisemitism-its-dangerous-usa-today/> (accessed July 19, 2026).
28. Nazarian S. Diversity, equity, and inclusion has no place in medicine — opinion. The Jerusalem Post 2023; December 30. Available at: <https://www.jpost.com/opinion/article-780128> (accessed April 16, 2026).
29. Nystrom A. StandWithUs NW presents antisemitism in the healthcare workspace event. Mercer Island Reporter 2025; July 31. Available at: <https://www.mi-reporter.com/2025/07/31/standwithus-nw-presents-antisemitism-in-the-healthcare-workspace-event/> (accessed April 16, 2026).
30. Office of Public Affairs, U.S. Department of Justice (DOJ). Justice department opens investigation into Lincoln Memorial University for discrimination against Jewish students. DOJ Press Release 2026; February 13. Available at: <https://www.justice.gov/opa/pr/justice-department-opens-investigation-lincoln-memorial-university-discrimination-against> (accessed April 16, 2026).
31. Poizner A. Politicized healthcare: rising harassment of diaspora Jews in medicine - opinion. The Jerusalem Post 2024; February 18. Available at: <https://www.jpost.com/diaspora/antisemitism/article-787461> (accessed April 16, 2026).
32. PR Newswire. Thousands of medical professionals from coast-to-coast form ‘American Jewish Medical Association.’ PR Newswire 2024; June 3. Available at: <https://www.prnewswire.com/news-releases/thousands-of-medical-professionals-from-coast-to-coast-form-american-jewish-medical-association-302162012.html> (accessed April 16, 2026).
33. Rohrer GA. Randy Fine wants South Florida dentist’s license suspended over antisemitic preaching. Florida Politics 2024; May 13. Available at: <https://floridapolitics.com/archives/674046-randy-fine-wants-south-florida-dentists-license-suspended-over-antisemitic-preaching/> (accessed April 16, 2026).
34. Saffren J. Doctor’s lawyers say he was mistreated due to his Judaism. Philadelphia Jewish Exponent 2024; May 8. Available at: <https://www.jewishexponent.com/doctors-lawyers-say-he-was-mistreated-due-to-his-judaism/> (accessed April 16, 2026).
35. Schuss DG. Is Hamas gaining a foothold in U.S. medicine? Aish.com 2024; February. Available at: <https://aish.com/is-hamas-gaining-a-foothold-in-u-s-medicine/> (accessed April 16, 2026).
36. Sofer B. Are American Jewish doctors being targeted by post-Oct. 7 antisemitism? – opinion. The Jerusalem Post 2025; February 7. Available at: <https://www.jpost.com/opinion/article-841065> (accessed April 16, 2026).
37. Song C. Federal agency to investigate Warren Alpert Medical School for alleged antisemitism. The Brown Daily Herald 2025; February 5. Available at: <https://www.browndailyherald.com/article/2025/02/federal-agency-to-investigate-warren-alpert-medical-school-for-alleged-antisemitism> (accessed April 16, 2026).
38. Starr M. Australian conference cancels Israeli doctor’s lecture after boycott calls. The Jerusalem Post 2024; March 4. Available at: <https://www.jpost.com/diaspora/antisemitism/article-790185> (accessed April 16, 2026).
39. Starr M. Australian nurse influencer causes uproar with Nazi salute video. The Jerusalem Post 2025; April 20. Available at: <https://www.jpost.com/diaspora/article-850818> (accessed April 16, 2026).
40. Starr M. Third of Jewish Ontario doctors consider leaving Canada over antisemitism - survey. The Jerusalem Post 2024; December 9. Available at: <https://www.jpost.com/diaspora/antisemitism/article-832582> (accessed April 16, 2026).
41. Sweeney E, Scales M. Dentist who allegedly tore down Israeli hostage posters fired from Boston practice. Boston Globe 2023; October 24. Available at: <https://www.bostonglobe.com/2023/10/24/metro/dentist-fired-israeli-posters/> (accessed April 16, 2026).
42. Trovato R. UCSF and UCLA medical school added to ongoing list of schools under antisemitism investigation. Daily Californian 2025; September 3. Available at: <https://www.dailycal.org/news/uc/ucsf-and-ucla-medical-school-added-to-ongoing-list-of-schools-under-antisemitism-investigation/article_3c96f21d-5eae-4cd6-a56a-4e481150b406.html> (accessed April 16, 2026).
43. U.S. Congress Committee on Education and the Workforce. @EdWorkforceCmte uncovers antisemitism at APA—Launches full investigation (press release). 2025; December 12. Available at: <https://edworkforce.house.gov/news/documentsingle.aspx?DocumentID=412932> (accessed April 16, 2026).
44. U.S. Department of Health and Human Services. HHS’ Civil Rights Office finds Harvard University in violation of federal civil rights law. HHS Press Release 2025; June 30. Available at: <https://www.hhs.gov/press-room/hhs-finds-harvard-in-violation.html> (accessed April 16, 2026).
45. U.S. House Committee on Energy and Commerce. E&C Republicans open investigation into allegations of antisemitism at UC San Francisco. 2024; August 1. Available at: <https://energycommerce.house.gov/posts/e-and-c-republicans-open-investigation-into-allegations-of-antisemitism-at-uc-san-francisco> (accessed April 16, 2026).
46. Whyte LE. U.S. to probe reports of antisemitic incidents at top medical schools. The Wall Street Journal 2025; February 4. Available at: <https://www.wsj.com/us-news/education/u-s-to-probe-antisemitic-incidents-at-top-medical-schools-a49e7ac8> (accessed April 16, 2026).

## Supplement C: Additional Sources Identified Through Hand-Searching, *n*=49.

These records were obtained by searching reference lists in the PubMed and ProQuest articles shown in the references of the main manuscript and in Supplements A and B, above.

1. Ancis J. Open letter to the American Psychological Association demanding accountability. Times of Israel 2025; February 25. Available at: <https://blogs.timesofisrael.com/open-letter-to-the-american-psychological-association-demanding-accountability/> (accessed April 16, 2026).
2. Ancis JR, Weisskirch RS. A tool for addressing antisemitism: The NASW Code of Ethics. J Hum Behav Soc Environ 2025;35:1443–9. <https://doi.org/10.1080/10911359.2025.2468369>
3. Antisemitism Policy Trust. Antisemitism in the Healthcare Sector Since October 7 23. London, UK: Antisemitism Policy Trust; 2025. Available at: <https://archive.jpr.org.uk/object-5590> (accessed July 15, 2026).
4. Beck AF, Solomon BS, Mogilner LN, et al. Standing up to antisemitism, together. Acad Pediatr 2025;25:1102547. <https://doi.org/10.1016/j.acap.2024.07.014>
5. Bellan B. Valedictory speech delivered to graduating medical students sets off storm of controversy. Jewish Post and News 2024. Available at: <https://jewishpostandnews.ca/faqs/rokmicronews-fp-1/valedictory-speech-delivered-to-graduating-medical-students-sets-off-storm-of-controversy/> (accessed April 16, 2026).
6. Bilger M. Lincoln Memorial med school accused of refusing to give Jewish students off for holidays. The College Fix 2026; February 16. Available at: <https://www.thecollegefix.com/lincoln-memorial-med-school-accused-of-refusing-to-give-jewish-students-off-for-holidays/> (accessed April 16, 2026).
7. Brosbe MS, Kaufman CC, Getzoff Testa E, Dimentstein K, Guttman-Lapin D, Rasooli T. Is it really that bad? Antisemitic experiences among Jewish mental health professionals. Am Psychol 2026; March 2, online ahead of print. <https://doi.org/10.1037/amp0001642>
8. Change.org. Demand accountability from our CIR Union. 2023; November 7. Available at: <https://www.change.org/p/demand-accountability-from-our-cir-union> (accessed April 16, 2026).
9. Chelouche T, Czech H, Fox MA, et al. Statement on the Lancet Commission on Medicine, Nazism, and the Holocaust. Lancet 2023;402:1816–17. <https://doi.org/10.1016/s0140-6736(23)02461-3>
10. Cohen B. Timeline: how attacks on Jews in Australia have been growing since Oct 7. Forward.com 2025; December 14. Available at: <https://forward.com/news/790237/australia-antisemitism-timeline-oct-7-bondi-beach/> (accessed April 16, 2026).
11. Cox C, Marlowe D. Antisemitism in social work: findings from an exploratory national survey. J Relig Spirituality Soc Work 2024;43:3–17. <https://doi.org/10.1080/15426432.2023.2289435>
12. Czech H, Hildebrandt S, Reis SP, et al. The Lancet Commission on Medicine, Nazism, and the Holocaust: historical evidence, implications for today, teaching for tomorrow. Lancet 2023;402:1867–940. <https://doi.org/10.1016/s0140-6736(23)01845-7>
13. Department of Health and Social Care, The Rt Hon Sir Keir Starmer KCB KC MP and The Rt Hon Wes Streeting MP UK. Government to tackle antisemitism and other racism in the NHS. Gov.UK 2025; October 16. Available at: <https://www.gov.uk/government/news/government-to-tackle-antisemitism-and-other-racism-in-the-nhs> (accessed April 16, 2026).
14. Deutch G. Chicago therapist who created blacklist of ‘Zionist’ therapists disciplined by state licensing body. Jewish Insider 2025; April 4. Available at: <https://jewishinsider.com/2025/04/jewish-therapists-zionist-blacklist-chicago-reprimand-heba-ibrahim-joudeh/> (accessed April 16, 2026).
15. Deutch G. Jewish organizations urge pediatricians to retract letter of support for Hamas doctor. Jewish Insider 2025; January 29. Available at: <https://jewishinsider.com/2025/01/jewish-organizations-urge-pediatricians-to-retract-letter-of-support-for-hamas-doctor/> (accessed April 16, 2026).
16. Donlevy C. Miami dentist fired after he was caught tearing down posters of Israeli hostages. New York Post 2023; October 18. Available at: <https://nypost.com/2023/10/18/miami-dentist-fired-for-tearing-down-israeli-hostage-posters/> (accessed April 16, 2026).
17. Edelson D. ‘Echoes of the Holocaust’: Professor speaks out against rising antisemitism on medical campuses. Ynet News 2024; November 2. Available at: <https://www.ynetnews.com/health_science/article/ryg8kagwjg> (accessed April 16, 2026).
18. Effinger A. OHSU investigates antisemitic comments allegedly made by nurse on social media. Willamette Week 2025; February 26. Available at: <https://www.wweek.com/news/2025/02/26/ohsu-investigating-antisemitic-comments-allegedly-made-by-employee-on-social-media/> (accessed April 16, 2026).
19. Frenk J. Initiative to Combat Antisemitism. UCLA Office of the Chancellor Messages 2025; March 10. Available at: <https://chancellor.ucla.edu/messages/initiative-to-combat-antisemitism> (accessed April 16, 2026).
20. Getzoff Testa E, Becker JA, Brosbe MS, et al. Widening the tent: Jewish inclusivity, antisemitism, and recommendations for action in pediatric psychology. Clin Pract Pediatr Psychol 2024;12:442–56. <https://doi.org/10.1037/cpp0000542>
21. Gilson G. International federation votes against expelling Israeli social workers’ union. The Jerusalem Post 2026; February 19. Available at: <https://www.jpost.com/diaspora/article-887180> (accessed April 16, 2026).
22. Hart J, Garlick M. What companies can learn from Boston’s success with Jewish ERGs. Staffing Industry Analysts 2025; April 30. Available at: <https://www.staffingindustry.com/editorial/staffing-stream/what-companies-can-learn-from-boston-s-success-with-jewish-ergs> (accessed April 16, 2026).
23. Harvard University. Presidential Task Force on Combating Antisemitism and Anti-Israeli Bias. 2025; April 29. Available at: <https://www.harvard.edu/wp-content/uploads/2025/04/FINAL-Harvard-ASAIB-Report-4.29.25.pdf> (accessed April 16, 2026).
24. Hauser, B. Inside a new MIT initiative that aims to offer solution to what it sees as widespread bias against Israeli scientists. Boston Globe 2025; August 4. Available at: https://www.bostonglobe.com/2025/08/04/metro/mit-kalaniyot/ (accessed April 16, 2026).
25. Jewish Federations of North America. Jewish Federations urge Congress to investigate healthcare antisemitism. 2024; December 18. Available at: <https://www.jewishfederations.org/blog/all/jewish-federations-urge-congress-to-investigate-healthcare-antisemitism> (accessed April 16, 2026).
26. Jewish Social Work Consortium. Biased curriculum and harassment reported at University of Illinois Chicago. 2026; March 30. Available at: <https://jewishsocialworkconsortium.com/news/> (accessed April 16, 2026).
27. Kahn BA. Standing against antisemitism in the dental workplace. Compendium of Continuing Education in Dentistry 2025; June 1. Available at: <https://compendiumlive.com/2025/06/standing-against/> (accessed April 16, 2026).
28. Katz D. The NHS must act now to stamp out bigotry. The Jewish Chronicle 2024; March 27. Available at: <https://www.thejc.com/opinion/the-nhs-must-act-now-to-stamp-out-bigotry-l3lpn78y> (accessed April 16, 2026).
29. Kazevman G. Hiding in Canadian medicine. Can Med Educ J 2024;15:118–19. <https://doi.org/10.36834/cmej.79584>
30. Kogan J. Report and testimony: antisemitism in social work. Jewish Fierce Compassion with Jen Kogan (Substack.com) 2025; September 15. Available at: <https://jenniferkogan.substack.com/p/new-report-documents-pervasive-antisemitism?r=m4xo6> (accessed April 16, 2026).
31. Lewak D. NYC doctor fired over disturbing anti-Israel posts denying Oct. 7 attacks: ‘Long live Hamas.’ New York Post 2025; March 29. Available at: <https://nypost.com/2025/03/29/us-news/mount-sinai-doctor-fired-for-posts-denying-october-7-attacks/> (accessed April 16, 2026).
32. McDonald P. Some Jewish patients reportedly hide identities as UCSF faces accusations of bias and activism. Campus Reform.org 2024; November 28. Available at: <https://www.campusreform.org/article/jewish-patients-reportedly-hide-identities-ucsf-faces-accusations-bias-activism/26920> (accessed April 16, 2026).
33. Patel DT, Yoon GE, Crimson Staff Writers. Health and Human Services Dept. to investigate Harvard Medical School over commencement protests. The Harvard Crimson 2025; February 5. Available at: <https://www.thecrimson.com/article/2025/2/5/hhs-investigation-hms-commencement/> (accessed April 16, 2026).
34. Pierre DJ. Three-quarters of Jewish students worldwide report concealing religious identity on campus, new survey finds. The Algemeiner 2025; September 16. Available at: <https://www.algemeiner.com/2025/09/16/three-quarters-jewish-students-worldwide-report> (accessed April 16, 2026).
35. Pope F. Revealed: how antisemitic bigotry surged through the NHS after the October 7 atrocities. Jewish Chronicle 2024; March 27. Available at: <https://www.thejc.com/news/revealed-how-antisemitic-bigotry-surged-through-the-nhs-after-the-october-7-atrocities-pq1zg98v> (accessed April 16, 2026).
36. Prince CJ. Med students’ antisemitic comments after Oct. 7 roil two prominent DC universities. Times of Israel 2023; December 19. Available at: <https://www.timesofisrael.com/med-students-antisemitic-comments-after-oct-7-roil-two-prominent-dc-universities/> (accessed April 16, 2026).
37. Roth S, Wald HS, Spence NZ, Oratz R, Schwartz DM. Hypocrisy of moral imperatives in the Israel–Hamas war. Lancet 2024;403:1542. <https://doi.org/10.1016/s0140-6736(24)00241-1>
38. Sibarium A. ‘Pedagogical Malpractice’: Inside UCLA Medical School’s mandatory ‘Health Equity’ class. Free Beacon 2024; April 24. Available at: <https://freebeacon.com/campus/pedagogical-malpractice-inside-ucla-medical-schools-mandatory-health-equity-class/> (accessed April 16, 2026).
39. Sousa-Meixell L, Weiss EL, Strassman JA, Schwartz SL, Marrus M. Antisemitism and social work: editorial introduction. J Hum Behav Soc Environ 2025;35:1347–52. <https://doi.org/10.1080/10911359.2025.2563013>
40. Strous M. Response to ‘An open letter: in solidarity with all children suffering in wars; to all who hold or share a concern for the wellbeing of children.’ S Afr Med J 2025;115:e3390. <https://doi.org/10.7196/samj.2025.v115i5.3390>
41. Swindle DM. UK doctor known for antisemitic posts arrested after violating bail, charged with inviting support for Hamas. The Algemeiner 2026; March 27. Available at: <https://www.algemeiner.com/2026/03/27/uk-doctor-known-antisemitic-posts-arrested-violating-bail-charged-inviting-support-hamas/> (accessed April 16, 2026).
42. Tobin JS. Don’t mourn the Holocaust while supporting the genocide of living Jews. Jewish News Syndicate 2026; January 26. Available at: <https://www.jns.org/column/jonathan-s-tobin/dont-mourn-the-holocaust-while-supporting-the-genocide-of-living-jews> (accessed April 16, 2026).
43. Tress L. ‘We don’t want no Zionists here’: Anti-Israel activists protest against New York City hospital. Times of Israel 2025; January 6. Available at: <https://www.timesofisrael.com/liveblog_entry/we-dont-want-no-zionists-here-anti-israel-activists-protest-against-new-york-city-hospital/> (accessed April 16, 2026).
44. Van As AB, Sidler D, Allema JH, et al. An open letter: in solidarity with all children suffering in wars; to all who hold or share a concern for the wellbeing of children. S Afr Med J 2025;115:e3044. <https://doi.org/10.7196/samj.2025.v115i3.3044>
45. Walberg T. Chairman Walberg investigates antisemitism at University of Illinois, UCSF, and UCLA Medical Schools. Press Release, U.S. House of Representatives 2025; August 25. Available at: <https://walberg.house.gov/media/press-releases/chairman-walberg-investigates-antisemitism-university-illinois-ucsf-and-ucla> (accessed April 16, 2026).
46. Walberg T. Letter to Dr Debra Kawahara regarding antisemitism at the American Psychological Association. Washington, DC: U.S. House Committee on Education and Workforce. 2025; December 12. Available at: <https://edworkforce.house.gov/uploadedfiles/apa_letter_12.10.2025.pdf> (accessed April 16, 2026).
47. Wald HS, Ehrenfeld JM. Studying the role of medicine in Nazism and the Holocaust to shape professional identity formation. Acad Med 2025;100:530. <https://doi.org/10.1097/acm.0000000000005988>
48. Wald HS, Reis SP, Lepicard E, Chelouche T, Fox MA. Rapid response to Gaza: doctors’ testimonies demand a permanent ceasefire by Abassi K. BMJ 2025. Available at: <https://www.bmj.com/content/389/bmj.r1176/rapid-responses> (accessed July 15, 2026).
49. Waldman-Levi A, Friedman Z, Falk-Kessler J, et al. Jewish students’ experiences of antisemitism in higher education settings and daily occupations. OTJR (Thorofare N J) 2026;46:493–500. <https://doi.org/10.1177/15394492251344525>
